# USER-CENTERED ANALYSIS OF A FITNESS APP FOR OLDER ADULTS: SENIOR FIT

**DOI:** 10.1093/geroni/igae098.2571

**Published:** 2024-12-31

**Authors:** Kathy Lee, Kate Hyun, Sabrina Haque, Kyle Henry, Troyee Saha, Jessica Cassidy, Soeun Jang, Christoph Csallner

**Affiliations:** The University of Texas Arlington, Arlington, Texas, United States; The University of Texas Arlington, Arlington, Texas, United States; The University of Texas Arlington, Arlington, Texas, United States; The University of Texas Arlington, Arlington, Texas, United States; The University of Texas Arlington, Arlington, Texas, United States; The University of Texas Arlington, Arlington, Texas, United States; University of Texas at Arlington, Arlington, Texas, United States; The University of Texas Arlington, Arlington, Texas, United States

## Abstract

Utilizing technology can greatly improve the health and well-being of older adults, especially those who prefer to age in place. Senior Fit, an augmented reality mobile application, was created specifically to assist older adults with various physical exercises. This poster presentation focuses on the specifics of the app, user feedback, and suggestions for designing apps aimed at enhancing the physical activity of older adults. The app features a simple login process, requiring only users’ first and last names along with their contact number. It provides access to 16 indoor exercise categories, including strength, cardio, and balance routines, as well as an outdoor walking feature utilizing GPS tracking to monitor distance and duration. Additional features include activity guidelines and progress tracking. We collected both quantitative and qualitative data from 20 older adults who used the app for 24 weeks. Quantitative data showed overall usage of the app, indicating the participants more frequently utilized the app for indoor activities than outdoor activities. Qualitative data identified significant potential for Senior Fit alongside several challenges. Our participants expressed a preference for automatic tracking akin to wearable devices and stressed the importance of accessing their progress data seamlessly integrated into their daily routines. On the other hand, many hesitated to use the camera-based features, raising usability concerns. These findings underscore the necessity of adhering to user-centered design principles and addressing the unique challenges older adults face in adopting app-based technology. By considering these insights, future mobile applications should address the specific needs of this demographic.

